# Contraceptive use and associated factors among sexually active female adolescents in Atwima Kwanwoma District, Ashanti region-Ghana

**DOI:** 10.11604/pamj.2019.32.182.15344

**Published:** 2019-04-12

**Authors:** Joyce Agyemang, Sam Newton, Isaac Nkrumah, Joyce Mahlako Tsoka-Gwegweni, Samuel Nambile Cumber

**Affiliations:** 1 Kwame Nkrumah University of Science and Technology, Accra, Ghana; 2 Kwame Nkrumah University of Science and Technology Hospital, Ghana & Garden City University College, Accra, Ghana; 3 Faculty of Health Sciences, University of the Free State, Bloemfontein, South Africa; 4 School of Nursing & Public Health, College of Health Sciences, University of KwaZulu-Natal Durban, South Africa; 5 Section for Epidemiology and Social Medicine, Department of Public Health, Institute of Medicine (EPSO), the Sahlgrenska Academy at University of Gothenburg, Gothenburg, Sweden; 6 School of Health Systems and Public Health Faculty of Health Sciences, University of Pretoria, Pretoria, South Africa

**Keywords:** Prevalence, contraceptive, female, adolescent, Ghana

## Abstract

**Introduction:**

Unintended pregnancies and adolescent childbearing are on the increase in Sub-Saharan Africa. In Ghana, 14% of adolescents aged 15-19 are already mothers or pregnant with their first child. Most of these pregnancies are associated with poor outcomes such as miscarriages, stillbirths, unsafe abortions and other complications that might result in infant or mortality. In addition, sexually-active adolescents (16-19 years) are at higher risk of contracting STIs. Evidence suggest that contraceptive use help reduce fertility rate and adolescent reproductive health. This study therefore sought to understand the magnitude and associated factors that influence female adolescents' use of contraceptive in the Atwima Kwanwoma District, Ghana.

**Methods:**

A descriptive and analytic cross-sectional study design was used for this study. Using a structured questionnaire, data were collected from randomly sampled 200 sexually active female adolescents; aged 16-19 for a three month period; June to September 2017. The questionnaire elicited data on the socio-demographic characteristics of respondents, their knowledge and perception, use of contraceptives and factors influencing their contraceptive use. Data were analyzed using STATA version 12.1 software.

**Results:**

Ninety-five percent of the respondents exhibited some knowledge about contraceptives, but this high knowledge did not translate into its use as the prevalence rate was 18%. Condom was the most widely used contraceptive (33%) and perceived side effects of contraceptives was found to be the main reason for not using the contraceptives (53.66%). Marital status and the participants who were staying with both parents were found to be associated with contraceptive use with their p-values of 0.023 and 0.002 respectively.

**Conclusion:**

Considering the fact that contraceptive knowledge does not necessarily translate into use, further studies (qualitative), are needed to understand why high knowledge levels are not associated with high usage patterns.

## Introduction

It is anticipated that, the world population will reach 8.5 billion in 2030, 9.7 billion in 2050 and 11.2 billion by 2100 [1]. Much of the population growth occurs in the least developed parts of the world, which is Sub-Saharan Africa and Asia. The additional 2.4 billion people expected to be added to the global population between 2015 and 2050; 1.3 billion will be added in Africa [1]. According to [2] Ghana's population increased by 30.4% from 18,912,079 in 2000 to 24,658,823 in 2010. This rapid population growth causes constraints on future economic growth and the ability of a country to provide for the welfare of its citizens and achieve its national development objectives. To help address the issue of rapid population growth, strategies need to be put in place to reduce the fertility rate. A major method of reducing fertility rate is through the use of contraceptives [3]. The Government of Ghana and its development partners as well as international NGOs have been implementing activities to improve the use of contraceptives for several decades. Although some progress has been made especially in the area of knowledge, a large number of Ghanaian female adolescents rarely use modern contraceptives [3]. Education on contraceptives need to be intensified among adolescents to enable them protect themselves from unplanned pregnancies, unsafe abortions and STIs. In Ghana, 14% of adolescents aged 15-19 are already mothers or pregnant with their first child [4]. Despite the numerous economic, social and health challenges of adolescent childbearing, several unmarried adolescents in the Atwima Kwanwoma District have two or more children and some others who use many kinds of concoctions to induce abortion. However, the incidence of unwanted pregnancy and STI acquisition among adolescents can be reduced by effective access and use of modern contraceptives [5]. With a very low (13%) contraceptive prevalence rate among female adolescents, the researcher therefore sought to understand the magnitude and the associated factors influencing contraceptive use among this population in the Atwima Kwanwoma District. In doing so, the female adolescents' knowledge about contraceptives, perception about contraceptive use and the factors affecting the utilization of contraceptives were determined. Effective application of this study's recommendations will improve the reproductive health of adolescent girls in the country, which will increase their contribution to the social and economic development of the country.

## Methods

A descriptive, analytical cross sectional study design was used in carrying out this study. The study was conducted in the Atwima Kwanwoma District, one of the 27 districts in the Ashanti region of Ghana. The district has four sub-districts and 58 communities. According to the District Health Management Team (DHMT), the district has a population of 103004 with a growth rate of 3% per annum. Most of the population are engaged in agriculture (62.6%). Health care services are being delivered in 11 health institutions in the district with a total capacity of one hundred and twenty-three health personnel. These institutions are five (5) health centres, two (2) Christian Health Association of Ghana (CHAG) or Mission Health facilities, one (1) private facility and three (3) Community Health Planning Services (CHPS) compounds The district has 54 outreach points where Reproductive and Child health (RCH) Services are rendered, including health promotion. A total of 200 female adolescents between the ages of 16-19 years who were identified as sexually active and were residents in the Atwima Kwanwoma District for at least 6 months recruited for the study. However, adolescents within the age group of 16 and 19 who were not engaged in sexual activity and those who did not consent to participate in the study were excluded. A simple random sampling technique was applied to select sexually active female adolescents between the age groups of 16 to 19. Respondents were selected irrespective of their level of education. Three (3) communities were randomly selected from each of the four Sub-districts and then another random sampling of ten already labeled households from each of the twelve (12) selected communities was done. Any selected household which was found not to have adolescents aged 16 to 19 was replaced by another randomly sampled household. Once a household was selected, all adolescents aged 16 to 19 who consented or whose parents consented were considered for the study. In selecting the communities, the names of all the communities in each of the sub-districts were written on pieces of paper and folded (a sub-district at a time). The folded papers were kept in a plastic container and were well shaken to adequately mix them up. A volunteer was made to pick one folded paper at a time and the names of those communities picked constituted the chosen communities for the study. This same process was used in selecting the households.

**Sample size:** The sample size was determined with the following factors in mind; estimated prevalence of contraceptive use among female adolescents in the Atwima Kwanwoma District which was 13%, the desired level of confidence which was pegged at 95% (a standard value of 1.96) and an acceptable margin of error which was 5% with a standard value of 0.05. Using the sample size calculation formula below, a sample size of 174 was calculated.

$$N=\frac{z^{2}\times pq}{e^{2}}$$

Where N = minimum sample size required; Z = confidence level at 95% p = estimated proportion of female adolescents using contraceptives q = 1-p e = margin of error at 5% N = (1.96)^2^ x 0.13(1-0.13) (0.05)^2^ N = 174 A non-response rate of 10% was calculated and added, that made the sample size 191 and this was rounded up to the nearest hundred. Therefore, a total sample size of 200 was used. A structured questionnaire containing closed ended questions was used to collect the data in twelve communities in the Atwima Kwanwoma District after it was pre-tested. The questionnaire had sections that elicited the socio-demographic characteristics of participants, their knowledge and perception about the use of contraceptives. Moreover, data on whether participants had ever used contraceptive, the type of contraceptive used and factors influencing their usage were also obtained. The research team after explaining how to answer the items on the questionnaire administered them to eligible participants who consented or assented to partake in the study. Participants who could read and write answered the items themselves. For those who could not, the questions were explained to them in a language they understand for them to answer. The questionnaire was however, pre-tested on ten (10) female adolescents at Deekrom, which is one of the communities in the Atwima Kwanwoma District. Data were entered into excel, imported into Stata version 12.1 and analyzed according to the research questions. Frequencies and proportions were performed to show the level of respondents' knowledge, their perception about contraceptives, use of contraceptive among them and factors influencing its use. However, a Chi-square test was used to determine the association between the dependent variable (contraceptive use) and respondent's socio-demographic characteristics. Level of significance was set at a P-value of 0.05. The sensitive nature of the topic is such that some respondents might not give candid answers. Moreover, the use of local language for those who could not read and write, may lead to misinterpretation of the questions. These limitations could lead to inaccurate results. Lastly, despite the training given to the research assistants, they could be bias in their questioning.

**Ethical considerations:** An Informed consent form was used to seek the consent of respondents and they were assured of confidentiality and complete anonymity. Ethical approval was sought from the Committee on Human Research, Publications and Ethics (CHRPE) of the Kwame Nkrumah University of Science and Technology and permission was sought from the District Health Management Team, Chiefs and Opinion leaders before the study commenced.

## Results

**Socio-demographic characteristics of respondents:** As indicated in the Table 1, of the 200 participants, all representing a 100% response rate were involved in the study. More than half of the participants 105 (52.5%) were aged 16-19 years, whereas the remaining 47.5% accounted for those aged 18-19 years. Out of the total number of respondents, 22.5% had no formal education. Most of the respondents (37.5%) were cohabiting with very few 12% had divorced/separated. Respondents who had one child made up the majority (42.5%). While majority (40.5%) responded staying with their partners, 10.5% stays with their father and 13% stays alone.

**Table 1:** Socio-demographic characteristics of the respondents

Variables	Frequency N (200)	Percent (%)
**Age**		
16yrs	48	24.00
17yrs	57	28.00
18yrs	49	24.50
19yrs	46	23.00
**Education**		
No school	45	22.50
Primary	51	22.50
JHS	64	32.00
SHS/Tech./Vocational	33	16.50
Tertiary	7	3.50
**Marital status**		
Single	41	20.50
Married	60	30.00
Divorce/Separated	24	12.00
Cohabiting	75	37.50
**Occupation**		
Artisan	53	26.50
Business	51	25.50
Farming	20	10.00
Student	18	9.00
Unemployed	58	29.00
**No of Children**		
None	48	24.00
One	85	42.50
Two	51	25.50
More than 2	16	8.00
**Religion**		
Christian	139	69.50
Muslim	43	21.50
Traditional	18	9.00
**Ethnicity**		
Akan	162	81.00
Ewe	10	5.00
Ga	9	4.50
Frafra	19	9.50
**Whom respondents stay with**		
Alone	26	13.00
Both parents	36	18.00
Father only	21	10.50
Mother only	36	18.00
Partner	81	40.50

**Respondents' knowledge on contraceptives:** There were 190 (95.0%) participants who could tell how they understood contraceptives and were also able to mention at least a method of contraceptive known and their benefits. Most of them (50.0%) deem it appropriate that contraceptive prevent unintended pregnancies as compared to minority of them (5.0%) who did not have any idea about contraceptive methods and their benefits. It was noted that condom as a method of contraceptive was known by the majority of the respondents (30%), followed by injectable (25.0%) whilst a few (5.0%) knew about IUD. Moreover, 39% of the respondents stated prevention of unintended pregnancy as a benefit of contraceptive whilst 15.0% said contraceptives help to plan the number of children one wants to have as detailed in Table 2.

**Table 2:** Respondents’ knowledge on contraceptives

Variables	Frequency	Percent (%)
**Ever heard of contraceptives**	N=200	
Yes	192	96.00
No	8	4.00
**Source of information**		
Health facility	47	23.50
Media	57	28.50
Parents	30	15.00
Peers	38	19.00
School	20	10.00
Not applicable	8	4.00
**Respondents Understanding of contraceptive**		
Drugs that help couples to have few children	26	13.00
Drugs that helps plan your life	30	15.00
Drugs that help prevent unintended pregnancy	100	50.00
Not applicable	10	5.00
**Contraceptive method known**	(N=200)	
Condom	60	30.00
IUD	10	5.00
Injectable	50	25.00
Jadelle	18	9.00
Pills	38	19.00
Sterilization	14	7.00
Not applicable	10	5.00
**Benefits of contraceptives by respondents**		
Prevents unintended pregnancy	78	39.00
Prevents STIs including HIV/AIDS	34	17.00
Helps to plan number of children one wants to have	30	15.00
Helps to space children	48	24.00
Not applicable	10	5.00

**Perception on contraceptive use:** More than half of respondents (52.0%) reported that use of contraceptives among adolescents does not promote promiscuity, followed by 33.0% who believe it does. As shown in Table 3 below, participants who perceived that contraceptives have health risks constituted the majority (70%) whilst 10% disagreed to that assertion. Regarding the process of acquiring a contraceptive, most of the respondents (47.0%) did not perceive the process of contraceptive acquisition as embarrassing. Also most of the respondents (39.0%) disagreed that contraceptives use is only the responsibility of women while (13.0%) were indecisive. With religion prohibition of contraceptive use, majority (39.0%) agree to it whilst 7.0% strongly disagree.

**Table 3:** Perception on contraceptive use

Variables	Frequency N (200)	Percent (%)
**Contraceptives for only adult married persons**		
Strongly agree	26	13.00
Agree	54	27.00
Neither agree nor disagree	26	13.00
Disagree	86	43.00
Strongly disagree	8	4.00
**Adolescents who use contraceptives are bad**		
Strongly agree	14	7.00
Agree	52	26.00
Neither agree nor disagree	30	15.00
Disagree	94	47.00
Strongly disagree	10	5.00
**Contraceptives have health risks (e.g. infertility)**		
Strongly agree	58	29.00
Agree	82	41.00
Neither agree nor disagree	40	20.00
Disagree	16	8.00
Strongly disagree	4	2.00
**Contraceptives have side effects e.g menstrual disorders**		
Strongly agree	82	41.00
Agree	58	29.00
Neither agree nor disagree	44	22.00
Disagree	14	7.00
Strongly disagree	2	1.00
**Process of acquiring is embarrassing**		
Strongly agree	12	6.00
Agree	48	24.00
Neither agree nor disagree	46	23.00
Disagree	86	43.00
Strongly disagree	8	4.00
**Contraceptives should be used by only women**		
Strongly agree	30	15.00
Agree	32	16.00
Neither agree nor disagree	26	13.00
Disagree	78	39.00
Strongly disagree	34	17.00
**Religion prohibits the use of contraceptives**		
Strongly agree	14	7.00
Agree	78	39.00
Neither agree nor disagree	30	15.00
Disagree	64	32.00
Strongly disagree	14	7.00
**Contraceptive use can cause cancer in women**		
Strongly agree	22	11.00
Agree	48	24.00
Neither agree nor disagree	100	50.00
Disagree	22	11.00
Strongly disagree	8	4.00

**Adolescents contraceptive use:** An overwhelming majority of the respondents (82.0%) did not use contraceptives. Also, among the respondents who use contraceptives, a significant proportion of them (33.3%) use condoms with 11.1% of respondents using pills. Out of the 164 respondents who do not use any type of contraceptives, majority (53.66%) stated that the side effects of contraceptive accounted for their non-usage whilst 3.66% attributed their non-usage to inconvenience associated with contraceptive use (Table 4).

**Table 4:** Usage patterns of contraceptive by adolescents

Variables	Frequency N (200)	Percent (%)
**Use of contraceptive**	(N = 200)	
Yes	36	18.00
No	164	82.00
**Type of contraceptive used**	(N = 36)	
Condoms	12	33.30
Injectables	10	27.80
Jadelle	10	27.80
Pills	4	11.10
**Where respondents acquire their contraceptives**	(N = 36)	
Hospital	22	61.10
Pharmacy	12	33.30
Friends	2	5.60
**Partners agreement to contraceptives use**	(N = 36)	
Yes	32	88.90
No	4	11.10
**Reasons for non-use of contraceptives**	(N = 164)	
Costly	10	6.10
Desire to become pregnant	14	8.54
Inconvenient to use	6	3.66
Infrequent sex	18	10.98
Lack of knowledge regarding method and source	10	6.10
Method failure	10	6.10
Religious prohibition	8	4.88
Side effects	88	53.66

**Factors influencing adolescent contraceptive use:** From Table 5 below, participants who could state where contraceptive can be accessed constituted the majority (85%). Regarding religious approval on the usage of contraceptives, participants who reported that religion permits the use of contraceptives formed the majority (58.0%). Majority of respondents (67.0%) agreed that their cultural practices in the community do not accept the use of contraceptives. As high as 63% of the respondents do not discuss reproductive health issues at home. More than half of the respondents (77.0%) believe that providing contraceptive services within reasonable time encourage its use. An overwhelming majority (80.0%) stated that free contraceptives will motivate people to use it. Again, majority of respondents (54.0%) agree that adolescents fear of being ridiculed by peers for being promiscuous if they use contraceptives, whilst 18.0% disagree to that assertion.

**Table 5:** Factors influencing contraceptive use

Variables	Frequency N (200)	Percent (%)
**Do you know where contraceptives can be accessed**		
Yes	170	85.00
No	30	15.00
**Whether the source of contraceptives are reachable**		
Yes	144	72.00
No	56	28.00
**Acceptance by one’s religion to use contraceptives**		
Yes	116	58.00
No	84	42.00
**Acceptance of the use of contraceptives by ones culture**		
Yes	66	33.00
No	134	67.00
**Do you discuss Reproductive health issues at home**		
Yes	74	37.00
No	126	63.00
**Providing services within reasonable time encourage the use of contraceptives**		
Yes	154	77.00
No	46	23.00
**Will free contraceptive services motivate you to use**		
Yes	160	80.00
No	40	20.00
**Providers of contraceptives are friendly**		
Strongly agree	42	21.00
Agree	102	51.00
Neither agree nor disagree	38	19.00
Disagree	12	6.00
Strongly disagree	6	3.00
**Providers encourage adolescents to use contraceptives**		
Strongly agree	40	20.00
Agree	110	55.00
Neither agree nor disagree	38	19.00
Disagree	12	6.00
Strongly disagree	0	0.00
**Adolescents fear that their peers will ridicule them**		
Strongly agree	24	12.00
Agree	104	52.00
Neither agree nor disagree	36	18.00
Disagree	36	18.00
Strongly disagree	0	0.00

**Association between socio-demographic characteristics of the respondents and the use of contraceptives:** Table 6 indicates the use of Chi-square to test for the association between socio-demographic characteristics of respondents and contraceptive use among sexually active female adolescents. From the findings, there were only two variables which were statistically associated with contraceptive use; marital status and the person whom respondent stays (alone, with partner or with parents) with p-values as 0.023 and 0.002 respectively.

**Table 6:** Association between socio-demographic characteristics of the respondents and the use of contraceptives

Variables	Usage of contraceptives		X^2^ (P-value)
	Yes, n (%)	No, n(%)	
**Age (years)**			1.7175 (0.633)
16	6 (16.67)	42 (25.61)	
17	11 (30.56)	46 (28.05)	
18	11 (30.56)	38 (23.17)	
19	8 (22.22)	28 (23.17)	
**Level of education**			5.2381 (0.264)
No school	6 (16.67)	39 (23.78)	
Primary	7 (19.44)	44 (26.83)	
JHS	11 (30.56)	53 (32.32)	
SHS/Technical/Vocational	10 (27.78)	23 (14.02)	
Tertiary	2 (3.50)	5 (3.05)	
**Marital status**			9.5677 (0.023)
Single	12 (33.33)	29 (17.68)	
Married	12 (33.33)	48 (29.27)	
Divorced/separated	6 (16.67)	18 (10.98)	
Cohabiting	6 (16.67)	69 (42.07)	
**Occupation**			6.8082 (0.146)
Artisan	9 (25.00)	44 (26.83)	
Business	15 (41.67)	36 (21.95)	
Farming	2 (5.56)	18 (10.98)	
Student	3 (8.33)	15 (9.15)	
Unemployed	7 (19.44)	51 (31.10)	
**No. of Children**			1.7496 (0.626)
None	10 (27.78)	38 (23.17)	
One	12 (33.33)	73 (44.51)	
Two	10 (27.78)	41 (25.00)	
More than 2	4 (11.11)	12 (7.32)	
**Religion**			0.2788 (0.870)
Christian	24 (66.67)	115 (70.12)	
Muslim	8 (22.22)	35 (21.34)	
Traditional	4 (11.11)	14 (8.54)	
**Ethnicity**			4. 6584 (0.199)
Akan	26 (66.67)	136 (82.93)	
Ewe	1 (2.78)	9 (5.48)	
Ga	3 (8.33)	6 (3.66)	
Frafra	6 (16.67)	13 (7.93)	
**The person whom the participant stays with**			17.0217 (0.002)
Alone	8 (22.22)	18 (10.98)	
Both parents	12 (33.33)	24 (14.63)	
Father only	0 (0.00)	21 (12.80)	
Partner	14 (38.89)	67 (40.85)	

## Discussion

The study assessed knowledge and perception about contraceptive and as well the factors that influence contraceptive use among sexually active female adolescents who were between the ages of 16-19 years at the Atwima Kwanwoma District.

**Respondents' knowledge on contraceptives:** The results of the study revealed that most of the participants (96%) had heard about contraceptives. This however, differs from findings of a study by [6] which assessed the knowledge and practice of contraception among school going children in India. From their study, the percentage of participants who were unaware of contraceptives was higher (51.0%) than those who had heard something about contraceptives (49.0%). It was also discovered in this study that most of the participants had high knowledge on contraceptives; as 95.0% of them could tell how they understood contraceptives and were also able to mention at least a method of contraceptive and some of its benefits. This probably may be because more than half of the participants (52%) had above Primary School education. It was noted that condom as a method of contraception was known by majority of the respondents (30.0%) followed by injectable (25.0%). This finding is similar to that of a study by [7] which assessed the knowledge of contraceptive methods among adolescents and revealed that condoms were the most common (72%) contraceptive method known by participants.

**Use of contraceptives:** The study showed that a small percentage of the participants (18.0%) had ever used contraceptive. This is far lower than the findings of a study by [8] at Asella Preparatory School in Ethiopia. The prevalence rate for contraceptive use among their participants was 61.0%. However, a study conducted by [9] revealed that contraceptive use among adolescents in Nairobi was lower than the current study (8.6%) even though family planning program was launched in Kenya in 1967. Among participants who used contraceptives, condom was the most used method (33.3%) followed by injectable and Jadelle (27.8% each), with pills being the least used method (11.1%). This might explain why majority of the respondents mentioned condom as the type of contraceptive method they know. The overall assessment of knowledge and use of contraceptive methods among the participants showed that the level of contraceptive knowledge did not translate into use as only 18.0% used contraceptives despite the high level of knowledge. This finding corresponds with a study by [10] which examined family planning needs of adolescents in predominantly rural communities in the central part of Ghana. They identified that female adolescents knowledge on contraceptive methods was high (87.7%) but utilization was low (17.9%) which indicates that their knowledge on contraception does not translate into usage. Majority of the participants (61.1%) who used contraceptive method acquired them from heath facilities probably because they are cheap at the health facilities than the pharmacy shops and also certain methods cannot be provided by non-health personnel. This result seems to be consistent with a study conducted by [11] which discovered that most female respondents believed the best place to access contraceptive was in the hospital. Most respondents who had ever used contraceptive (88.9%) confirmed that their partners agreed to contraceptive use.

**Perception on contraceptive use:** Among the 164 (82.0%) participants who did not use any method of contraceptive, majority (53.6%) stated side effects as the reason for non-use. This is in agreement with study on adolescents' willingness and intentions to use contraceptive in rural Ghana [12]. The study revealed that some adolescents sited side effects as a reason for not intending to use contraceptives. In this study, most of the participants disagreed to the fact that contraceptives are for only adult married persons, hence, most of them (49.0%) disagreed to the fact that adolescents who use contraceptives are labeled as bad. This finding contradicts that of [13] and [10] who found out in their studies that contraceptive use make adolescents promiscuous, therefore, when they use them they will be tagged as bad girls and will also make them careless. It was further identified in this study that, 70.0% of the participants perceived contraceptives to be associated with fibroid and infertility as well as side effects like menstrual disorders. Similar results was discovered by [14]. In their study, perceived health risks including effects on menstruation, weight and future fertility were shown to be primary barriers to contraceptive use.

**Factors influencing contraceptive use:** When factors influencing contraceptive use were examined, it revealed that majority of the participants (85.0%) knew where contraceptives could be accessed, whilst 72.0% stated the source as easily reachable. However, 80.0% of participants admitted that providing free or cheap contraceptives services will motivate its use. This presupposes that the use of contraceptives goes beyond accessibility, which agrees with a study conducted in the Upper East region of Ghana [15]. In that study, it was observed that, a person's socio-economic status could influence contraceptive use as women in a better socio-economic status could afford contraceptive services. Majority of the participants (58.0%) admitted that religion was not a barrier to contraceptive use. However, the lack of acceptance of contraceptive use by one's culture was revealed as a key hindrance (67.0%). A similar finding was found by [16] in a study aimed at determining the factors influencing contraceptive use in Sub-Saharan Africa. It was noted in their study that cultural factors such as men's role in making decisions in a relationship and pressure to bear children had influence on contraceptive use and women are unlikely to use contraceptives if their partners disagree or are being pressured to give birth. This might explain why most women are scared to practice contraceptives without the approval from their husbands because it could jeopardize their marriage. Providers' attitude is another predictor for the use of contraceptives in the study as 72.0% of participants admitted that providers of contraceptive services were friendly to them and 75.0% also stated that providers encourage adolescents to use contraceptives. This finding differs from that of [17] who assessed the attitude of healthcare providers towards providing contraceptives to unmarried adolescents. In that study, it was concluded that healthcare providers had negative attitudes towards provision of contraceptives to unmarried adolescents as 57.5% of the health care providers had the opinion that use of contraceptive among the adolescents promotes sexual promiscuity.

**Association between socio-demographic factors and contraceptive use:** Respondents' educational level, occupation, religion, ethnicity and number of children they have were also found not show any significant association with contraceptive use in this study. This differs from [**15**] study which assessed determinants of contraceptive use and revealed that level of education, socio-economic status and parity of respondents were associated with contraceptive use. However, participants' marital status was found to have an association with contraceptive use (p-value = 0.023). It therefore appears from the study that those participants who were single were most likely to use contraceptives. The reason for this is likely to be that majority (37%) knew the use of contraceptives help prevent unwanted pregnancies. Similarly, a study by also showed an association between these two variables (marital status and use of contraceptive). Moreover, the study also found that the person whom the respondent stays with had a significant association with contraceptive use (p-value = 0.002). Thus those participants who were staying with both parents were most likely to use contraceptives. This may be as a result of fear of the participants not wanting their parents to see them pregnant.

## Conclusion

The study revealed that the prevalence rate of contraceptives among sexually active female adolescents at the Atwima Kwanwoma District is 18% although there was high knowledge of contraceptives among them. Among respondents who use contraceptives, condom was the method mostly used and the media was cited as the most common source of information on contraceptives. Perceived side effects were the major contributing factor for non-use of contraceptives. Age, level of education, occupation, number of children, religion and ethnicity showed no significant association with contraceptive use, while variables such as marital status and the person whom respondents stay with were found to be associated with contraceptive use with their p-values being 0.023 and 0.002 respectively.

**Recommendations:** Base on the findings from the study, the following recommendations were made: the District Health Directorate should collaborate with the Ghana Media Commission to promote use of contraceptives using the media such as radio and television; midwives, Community Health Nurses and other health care providers should be motivated to reach out to the people in the community to provide counselling and education on healthy birth spacing, address misconceptions and modern contraceptive methods; parents should also be open to discuss adolescent health issues at home; further research (qualitative) should be undertaken to understand why high knowledge levels are not associated with high usage patterns.

### What is known about this topic

- Contraceptives use is low in Sub-Saharan Africa;
- Knowledge on contraception does not translate into usage; high knowledge but low usage among adolescents;
- Side effects such as uterine fibroids, inability to conceive and marital status are known to influence adolescents from contraceptive use.

### What this study adds

- More than half of sexually active females reported that the use of contraceptives among adolescents does not promote promiscuity;
- Provider's attitude (friendly ones) encourages the use of contraceptives;
- Adolescents who stay with both parents appear to use contraceptives the most.

## Competing interests

The authors declare no competing interests.
